# Improving active case finding for tuberculosis in South Africa: informing innovative implementation approaches in the context of the Kharitode trial through formative research

**DOI:** 10.1186/s12961-017-0206-8

**Published:** 2017-05-30

**Authors:** Deanna Kerrigan, Nora West, Carrie Tudor, Colleen F. Hanrahan, Limakatso Lebina, Reginah Msandiwa, Lesego Mmolawa, Neil Martinson, David Dowdy

**Affiliations:** 1 Department of Health Behavior and Society, Johns Hopkins Bloomberg School of Public Health, 624 N. Broadway, Hampton House 257, Baltimore, MD 21205 United States of America; 2 Department of Epidemiology, Johns Hopkins Bloomberg School of Public Health, 615 N. Wolfe Street, Room E6532, Baltimore, MD 21205 United States of America; 3 International Council of Nurses, 3 Place Jean Marteau, 1201 Geneva, Switzerland; 4 Department of Epidemiology, Johns Hopkins Bloomberg School of Public Health, 615 N. Wolfe Street, Room E6031, Baltimore, MD 21205 United States of America; 5 Perinatal HIV Research Unit, University of Witwatersrand, Johannesburg, 2000 South Africa; 6 Department of Epidemiology, Johns Hopkins Bloomberg School of Public Health, 615 N. Wolfe Street, Room E6531, Baltimore, MD 21205 United States of America

**Keywords:** Tuberculosis, South Africa, Active case finding, Incentives, Household, Clinic

## Abstract

**Background:**

Tuberculosis (TB) is the leading infectious killer worldwide, with approximately 1.8 million deaths in 2015. While effective treatment exists, implementation of active case finding (ACF) methods to identify persons with active TB in a timely and cost-effective manner continues to be a major challenge in resource-constrained settings. Limited qualitative work has been conducted to gain an in-depth understanding of implementation barriers.

**Methods:**

Qualitative research was conducted to inform the development of three ACF strategies for TB to be evaluated as part of the Kharitode cluster-randomised trial being conducted in a rural province of South Africa. This included 25 semi-structured in-depth interviews among 8 TB patients, 7 of their household members and 10 clinic health workers, as well as 4 focus group discussions (2 rural and 2 main town locations) with 6–8 participants each (*n* = 27). Interviews and focus group discussions explored the context, advantages and limitations, as well as the implications of three ACF methods. Content analysis was utilised to document salient themes regarding their feasibility, acceptability and potential effectiveness.

**Results:**

Study participants (TB patients and community members) reported difficulty identifying TB symptoms and seeking care in a timely fashion. In turn, all stakeholder groups felt that more proactive case finding strategies would be beneficial. Clinic-based strategies (including screening all patients regardless of visit purpose) were seen as the most acceptable method based on participants’ preference ranking of the ACF strategies. However, given the resource constraints experienced by the public healthcare system in South Africa, many participants doubted whether it would be the most effective strategy. Household outreach and incentive-based strategies were described as promising, but participants reported some concerns (e.g. stigma in case of household-based and ethical concerns in the case of incentives). Participants offered insights into how to optimise each strategy, tailoring implementation to community needs (low TB knowledge) and realities (financial constraints, transport, time off from work).

**Conclusions:**

Findings suggest different methods of TB ACF are likely to engage different populations, highlighting the utility of a comprehensive approach.

**Trial registration:**

Clinicaltrials.gov (NCT02808507). Registered June 1, 2016. ﻿The participants in this formative study are not trial participants.

## Background

Tuberculosis (TB) is the leading single-agent cause of death by infection worldwide, with approximately 1.8 million people having died from TB in 2015 [1]. Additionally, 10.4 million people experienced TB-related morbidity that same year [1], and yet only 6 million new cases of TB were reported to WHO, indicating that over one-third of new cases in 2015 were undiagnosed or unreported [1]. Despite the lack of definitive evidence that systematic screening for TB leads to epidemiological impact, screening persons at high risk for TB has increasingly become a major global health priority, as it ensures that those with TB receive prompt treatment and may reduce ongoing transmission [2–5].

South Africa carries a significant amount of the global TB burden, with an estimated 450,000 new cases in 2014 [1]. However, only 306,000 cases were notified that year, indicating a 68% case detection proportion for the country overall, thus highlighting critical gaps in finding and treating all people with TB [1]. South Africa’s National Strategic Plan on HIV, TB and sexually transmitted infections emphasises active case finding (ACF) of TB, including TB screening among adult clinic attendees and contact investigation, as one of the two leading TB sub-objectives [6]. Details regarding the most effective approaches to achieve these objectives, however, remain sparse, demonstrating the importance of formative research to guide the implementation of these key policy priorities.

Multiple studies have documented the importance of moving beyond the clinic into households and communities to find active TB cases in order to overcome structural barriers preventing access to clinical care services among those in need [7–12]. However, operationalising these ‘community-based’ approaches has been challenging given ongoing TB-related stigma and the logistical and financial constraints of public health programmes [13–15].

To overcome such barriers, referral- and incentive-based approaches are being tested in some settings [16, 17]. For example, index cases may be offered financial incentives to refer their family members for TB screening, or community health workers may be given cash transfers conditional on their ability to recruit members of the community for TB screening. Such incentives can help mitigate the cost of TB disease, particularly in the context of the global priority to eliminate catastrophic costs related to TB [1, 18, 19]. However, these incentives can carry substantial costs to the health system and should only be recommended if found to be effective and cost-effective – evidence for which is currently lacking, particularly in resource-constrained rural African settings [20–22].

‘Kharitode TB’ is a randomised trial assessing the comparative yield and cost-effectiveness of three different ACF strategies (clinic-, household- and incentive-based) across 56 rural public clinics in two districts of the Limpopo province of South Africa over a period of 18 months. During a formative phase of the project, we utilised qualitative research methods to explore the feasibility and acceptability of each of these ACF approaches to inform their optimal implementation within the Kharitode trial.

## Methods

From October 2015 to January 2016, we conducted 25 semi-structured in-depth interviews (IDIs) and 4 focus group discussions (FGDs) (*n* = 27 participants) in the two districts (Vhembe and Waterberg) where the Kharitode trial is taking place. The IDIs included three types of adult participants (18 years of age or older), namely primary healthcare clinic workers (*n* = 10), TB patients (*n* = 8) and household members of TB patients (*n* = 7). For the IDIs, healthcare clinic workers who regularly care for TB patients were purposively selected. TB patients were recruited purposively with the help of clinic personnel, while TB patients assisted in recruiting their household members. Household members included both male and female partners/spouses and siblings of TB patients.

Among the 25 IDI participants, 16 were women and 9 were men. We stratified the sample by location, with 13 participants from a village setting and 12 from a main town. IDI participants ranged in age from 24 to 83 years, with a mean age of 41.5 years. FGDs were conducted with TB patients, recruited purposively using recommendations from clinic personnel, from both rural and town locations in the same districts. FGD participants were a mix of men (*n* = 15) and women (*n* = 12), and participants ranged in age from 23 to 59 years, with a mean of 36.9 years. All data was collected by Kharitode study staff trained in qualitative methods and techniques and fluent in the languages spoken in the area (English, Venda, Sipedi or Tsonga).

Using a topical field guide, IDIs and FGDs explored the following domains of interest: understanding of and experiences with TB symptoms and their detection, the TB diagnostic process, clinic experiences and perceived quality of TB care, and the perceived benefits (e.g. reduced symptom burden, reduced transmission, confidentiality) and potential negative consequences (e.g. stigma, inconvenience, over diagnosis) of each of the ACF strategies to be included in the trial. Participants were asked to reflect on how each of the three ACF strategies might be best operationalised to improve its feasibility, acceptability and effectiveness. Participants were also asked to rank each of the three strategies in terms of their perceived level of effectiveness to identify active TB cases. Interviewers described the three strategies to participants as indicated below (Table 1), explaining the setting and approach for each strategy.

**Table 1:** Active case finding strategies

Clinic	Household	Incentives
The clinic strategy would include screening everyone coming into primary care clinics for tuberculosis (TB) symptoms, and if they have one or more, testing them for TB	The household strategy would involve sending healthcare workers to the homes of new TB cases and testing everyone in the household for TB	The incentive strategy would involve giving new TB cases coupons to give to their friends, family and people who they spend time with whom they think could have TB; the TB case and the referred contact would both receive some form of an incentive if that person came to the clinic to be checked for TB

All IDIs and FGDs were conducted in a given participant’s preferred language. The IDIs and FGDs were audio-recorded and transcribed, and translated into English as necessary. Thematic content analysis was used to approach the textual data [23]. Coding was structured around pre-defined as well as emergent domains, including the context, feasibility, acceptability and potential effectiveness of the strategies explored. Code output was synthesised and utilised to identify salient themes (e.g. TB awareness, TB/HIV stigma, etc.) within each topical domain of interest across each population group and method. Illustrative quotes were selected to represent participant views around each strategy. Texts were coded using the qualitative data analysis software, ATLAS.ti^©^.

## Results

### Barriers to recognising TB and seeking care

Participants described a combination of individual (lack of TB knowledge), social (TB stigma) and structural factors (distance, time and lack of money for transportation to the clinic) impacting their ability to recognise and seek care for TB in a timely manner. Most TB patients interviewed described having flu-like symptoms (e.g. cough, fatigue, weakness, lack of appetite, weight loss, fever, sweats) for an extended period of time (several months) and not knowing why they were so sick, frequently resulting in being taken to the hospital via ambulance because they were so ill, and only afterward being diagnosed with TB. Clinic healthcare workers also noted that patients commonly mistook their TB symptoms for the flu, causing care-seeking delays.

While there were many patients with TB and their family members that did understand that the symptoms they had could be linked to TB, they also had other theories, such as exposure to dust in the home or in work environments (mines or timber factories), as to why they may have those symptoms. Participant reports also indicated significant fear of being diagnosed with TB or potentially with HIV, which shared similar symptoms according to participants, due to ongoing stigma and discrimination. One healthcare worker described these awareness and stigma-related concerns below:

“*I am not sure if is stigma or what, but the problem is that the patients don’t want to open up and they don’t want to be diagnosed with TB. They are afraid of being screened which I think maybe is because of the little knowledge which might mean that we are not giving enough health education the danger of TB. Nowadays people associate TB with HIV, so they think that if they can be diagnosed with TB people will also think that they are HIV positive. Also most of the people do not want to know their HIV status, so they are afraid if they are to be diagnosed with TB they will also be tested for HIV. They are afraid of knowing. As a result of the above factors it causes them to be diagnosed late and causes them to delay even the treatment. This is according to my knowledge. They also don’t take the symptoms serious, they just assume that is a flu.*” (Nurse, male, 29 years old)

### Clinic-based screening

Some participants reported having come to the clinic for other matters (pregnancy, flu, etc.) and were asked to test for other conditions such as HIV and TB, although this was not reported to be systematically performed. In turn, the idea of being approached at the clinic for TB screening seemed somewhat normal to many participants, as relayed below:

“*Clinics these days, if I get a headache, and they take my blood, they will be checking everything that I could have. If they find I don’t have TB, they find that I don’t have diabetes, they find that I don’t have this, you see? There is no problem. But if they check, when the machines they use to collect blood is there. If they check and that machine says there is AIDS. It tells you if you are negative or positive. They check for everything at the clinic these days. It’s what the government says, people must not leave if they have not been checked for everything. So we must come to the garage of doctors. Doctors are our garages. We are here to be fixed; you have to check every wire.*” (TB patient, male, 56 years old)

Participants were clear, however, that to not generate or reinforce stigma, all people should be screened, not just certain types of people (e.g. those with HIV, etc.). Additionally, several people commented that not all people will agree to be screened. However, most felt that people should “*respect the nurses*” and that it was for their own good to be checked, whether it be for TB, HIV or “*sugar diabetes*”. Participants noted that if someone is attending the clinic, they are open to the medical system and the idea of being checked. On the other hand, some participants noted that not everyone goes to the clinic, including those who tend to access traditional and faith-based healers. However, some noted that public clinics may be limited in their ability to realistically conduct TB screening of all patients given human resource constraints. Healthcare workers re-enforced this concern, indicating that, while they always tried to educate and screen patients, they are often short-staffed, which limits systematic implementation. Participants also raised concerns about TB screening leading to longer clinic visits and wait times.

### Household case finding

When asked about a strategy to test the household members of a person with TB, about half the participants expressed openness to this idea, indicating that it allowed for people to stay in the “*comfort of their own home*” and avoid travel time and costs. Several participants (both TB patients and household members) commented that an advantage to a household strategy would be that it would facilitate access to screening for those who might be elderly, very ill, and/or unable to come to the clinic for other reasons. Some also saw the potential for more discretion with household visits given the stigma concerns, as described above, regarding clinic attendance and in the quote below:

“*I think door-to-door* [is preferable] *because people are afraid of coming to the clinic and they feel ashamed and embarrassed. So if the nurse comes to the house and screens them at home, it is better than coming to the clinic because no one will see you that you haven’t screened.*” (FGD participant, TB patient)

However, many participants advised that the TB index case should ensure that the members of their household were made aware in advance of such a household visit and were generally in agreement in order to avoid potential problems. Both TB patients and household members reinforced the idea that there should be no “*unannounced*” visits and visits should not be forced upon people. Additionally, participants suggested the local tribal chief should be consulted and his support sought to help with overall community acceptance. They also highlighted that health workers should be trained to be respectful and not “*scold*” or “*crucify*” family members for not getting tested or seeking care earlier.

Despite support for the strategy among some study participants, others felt less comfortable with the household approach and warned that such visits could be associated with stigmatised conditions such as HIV, and lead to “*gossip*”, “*embarrassment*” and/or “*rumours*”. The idea that household visits are for “*people who are very sick*” was mentioned by several participants and some reported that this could be interpreted as them having HIV. Some patients even indicated that, despite knowing that they would lose a whole day’s time and incur transport costs to attend the clinic for screening, they would prefer that over the household strategy in order to avoid the potential stigma and gossip in their communities.

“*When they do house visit people will be gossiping about the particular person who has been visited by the nurses. They say that bad has become worse. They will think that the nurses are visiting because that person is bed ridden. People will have rumours about me as a patient, so is better for me to come to the clinic than having nurses coming to my house.*” (TB patient, female, 83 years old)

There were mixed views on whether health workers should potentially come in unmarked cars or in plain clothes versus uniforms for such home visits. However, most felt that they should come in official cars and uniforms in order to reassure community and household members that they were “*legitimate*” and to command respect and professional dignity. Safety was also noted as a key point by healthcare workers.

Healthcare workers shared concerns about the household strategy and added that, while one may be able to diagnose some cases in the home, patients must still attend the clinic to receive treatment and thus, clinic attendance is still preferred. Healthcare workers also noted that some mobile outreach (using civic leaders, Chiefs, etc.), school and door-to-door TB screening campaigns have been used in the past and that they had often not used marked cars when doing such outreach. Additionally, health workers noted the challenges of reaching more remote and rural areas versus those near the clinic.

### Material incentives

All participants agreed that most people seeking clinical care in the public health sector, including those receiving TB care and treatment, were generally in need of financial support and resources, and in turn incentives would be welcome from that perspective. Money and food or food vouchers were all seen as potentially useful incentives, with some forms of incentives being more preferable to certain demographic groups. For example, participants suggested that youth may prefer cell phone airtime, whereas women or people with families to feed may prefer food or food coupons over other types of incentives.

However, many participants felt that the idea of incentivising health and healthcare was somewhat counterintuitive in that it was not clear why people would need to be paid to do something that is ultimately to their own benefit. Others worried about the sustainability of such an approach or that some might try and manipulate the system to their benefit. Several questioned whether the government would be able to implement such an approach given the current lack of resources in public clinics. Additionally, though less commonly voiced, there were some fears of incentives appearing to be bribes or potentially coercive on the part of healthcare workers. Others felt that many people would not want to disclose their TB to others to motivate them to come to get screened.

In general, many participants commented that overall “*people love free things*” and that “*money is always number one*” (in terms of preference across the strategies) and that people would welcome some form of an incentive to recognise their time and struggles. Though amounts varied across participants, on average 200 South African rand (approximately 14 US dollars) was suggested as the incentive amount that should be given to a TB patient for each contact they convince to screen for TB. For the contact of a TB patient who presents for TB symptom screening, 125 South African rand (approximately 9 US dollars) was the average amount recommended. As one healthcare worker relayed:

“*I am choosing this strategy because there is no one person who does not want to be compensated. Once a person leaves their house to come to the clinic is because they are hoping and willing to improve their health, sometimes you will even find that they even borrowed the money which they have used for transport to the clinic. There are those people who are not even aware of the disease they don’t even have time to come to the clinic. Even if they can be called to come for screening, they cannot come. But if we are telling them that we are giving money to those people who are screening for TB, I believe they can come in multitude. And also each and every one will want to be screened. So unlike the strategy which is clinic based, how can we reach to those who won’t be coming to the clinic? So with this one everyone will want to come to the clinic. Because people only comes to the clinic when they are sick or when they are in need of medical care do you understand? Our main idea here is getting more people screen for TB the best way possible and as many people as possible. So to make this possible we have to use something that can attract everyone, so everyone will come.*” (Nurse, male, 29 years old)

### Alternative strategies: culturally appropriate and community-based approaches

In addition to the three strategies presented to participants, many offered their own thoughts about alternatives that might be effective. The idea of using community-based and culturally appropriate strategies to mobilise interest and participation in TB screening was seen as important across participant types. In particular, the use of respected leaders of the community, like Chiefs and civic leaders, to call the community to campaigns and events where groups of people could be screened was seen as highly acceptable. For example, as one household member conveyed when asked what other strategies could be effective at diagnosing new cases, the authority and support of the Chief could be essential:

“*When the nurses as they are, can go to the Chief’s place and the people of the community come out, and agree to get help, and agree and accept what the nurses are telling them….because isn’t it that at the Chief’s place they will say, ‘There is an urgent gathering, there is an issue, people from the clinic want to talk to people’. In such a way that if they were to go there, they would be helped. And they are good at convincing, and that person will feel that they also need to get checked.*” (Household member, female, 43 years old)

FGD participants also reinforced the idea that it may be important to go beyond “*door-to-door*” household approaches that targeted one house at a time given resource constraints and privacy concerns. They supported integrating more community-wide approaches as the following quote from a FGD participant indicated:

“*If they conduct campaigns at schools we will come because we will see in multitude everyone getting screened. So house-to-house will have problems because people will be even embarrassed of the healthcare workers coming to their houses and screen for TB.*” (FGD participant, TB patient)

## Discussion

This qualitative study informed the design of a cluster-randomised trial of different TB ACF strategies being conducted in rural South Africa. Its findings have implications for both the Kharitode study and the literature on ACF for TB in rural settings. In sub-Saharan Africa, 60% of the population still lives in rural areas [24], yet the majority of existing ACF research in sub-Saharan Africa has focused on urban settings and thus may not generalise to rural settings where high burdens of TB exist [21–23]. Overall, while participants described the potential advantages and disadvantages as well as preferences related to the three ACF strategies presented to them, they were often positive about the role that each might play in a comprehensive approach. In general, participants (particularly TB patients and their household members) felt that anything that could be done to assist them, their families and their communities to address TB would be seen positively and be well received. Healthcare workers also felt that the different strategies could be combined since aspects of each would work well for some, but not others.

In turn, their inputs were less oriented to whether one strategy should or should not be performed, but rather how to implement each of the strategies to optimise their success. Participants often struggled to distinguish what would be preferred from what would be effective. Data from existing studies show high acceptability of community-based ACF [14, 25]. Among study participants, many felt the household strategy could be effective in finding cases, but may not be appealing or acceptable to some in the community.

Additionally, participants also indicated the importance of mobile screening. A study in Zimbabwe found that a mobile screening approach yielded higher rates of TB case detection compared to a door-to-door approach [26]. Participants also felt community-based events at the local Civic, Royal, *Induna* (Traditional Leader) or Chief’s house or other culturally respected and valued stakeholder or organisation venues would be appropriate and well received. Such community-based mobilisation and events were seen to facilitate access to diagnosis and treatment, and could potentially complement household screening. The need for strategies that move outside of healthcare facilities to address barriers to care-seeking and treatment for TB has also been documented in other studies [27–29]. However, support for clinic-based case finding was voiced across all participant types. This aligns with findings from two existing studies in high TB burden regions indicating that screening individuals presenting for clinical care for any reason may yield a substantial number of new TB cases [30, 31].

This study has several limitations, including its cross-sectional and exploratory nature, thus inhibiting documentation of how these complex and dynamic social processes related to TB diagnosis and treatment may work and change over time and across settings. However, the use of triangulation, both in terms of participant type (TB patient, household member, and clinic healthcare worker) and methods (IDIs and FGDs), allowed for nuanced insights regarding both the potential strengths and limitations of each approach. It is also important to note that, while participants may have recommended specific monetary incentive levels, this should not be perceived as a policy recommendation (unless such incentive levels could be linked to clinical or epidemiological outcomes).

## Conclusion

Overall, participants felt that each of the three ACF methods explored would have pros and cons, and that each could identify unique populations of people with TB. Clinic-based approaches had the advantage that people were already motivated to seek clinical care, and that screening for other diseases beyond what one came to the clinic for, was now becoming commonplace in South Africa (e.g. HIV, diabetes, etc.). However, given the barriers to care-seeking, including transport, time off and financial burden, participants acknowledged the need to move beyond the clinic to the household and community. In turn, household screening was seen as one way to fill this gap, and reach “*harder to reach*” populations. Incentives were also understood to have a potential role given the consensus that financial challenges were widespread in rural South Africa; food, in particular, would be a particularly salient motivator to TB screening. Participants cautioned that, with each strategy, there is a need to be aware of and work to reduce TB (and related HIV stigma) stigma by working closely with community leaders and members to protect confidentiality and human rights.

Taken together, these findings suggest that a comprehensive, multi-pronged approach is necessary to reach distinct populations within a given geographic setting, as the needs of different groups are not likely to be met by one specific TB ACF approach or strategy. While awaiting the findings from the Kharitode study, these formative results can help inform national and district-level policy by emphasising the acceptability of TB ACF and the need for a comprehensive approach to effectively find TB cases (a high-level health priority) in rural settings.

## Abbreviations

ACF: active case finding
FGD: focus group discussion
IDI: in-depth interview
TB: tuberculosis
